# The Respiratory Burst of Human Granulocytes Is Mostly Independent of Potassium

**DOI:** 10.3390/biom15101362

**Published:** 2025-09-25

**Authors:** Iryna Mahorivska, Martin Geltinger, Gustavo Chaves, Sebastian Lobmann, Martin Jakab, Katharina Helm, Boris Musset

**Affiliations:** 1 Center of Physiology, Pathophysiology and Biophysics, Paracelsus Medical University, 90419 Nuremberg, Germany; iryna.mahorivska@klinikum-nuernberg.de (I.M.); gustavo.chaves@pmu.ac.at (G.C.); sebastian.lobmann@klinikum-nuernberg.de (S.L.); 2 Gemeinschaftspraxis Dres. Wiesheu/Buckl/Paul/Popp, 84032 Landshut, Germany; 3 Clinic of Gastroenterology, Hepatology and Endocrinology, Klinikum Nürnberg, Paracelsus Medical University, 90419 Nuremberg, Germany; 4 Center of Physiology, Pathophysiology and Biophysics, Paracelsus Medical University, 5020 Salzburg, Austria; martin.jakab@pmu.ac.at (M.J.); katharina.helm@pmu.ac.at (K.H.)

**Keywords:** potassium, voltage-gated proton channel, NADPH oxidase, osmolality, respiratory burst, leucocytes

## Abstract

Reactive oxygen species (ROS) are among the most effective tools of the innate immune response against pathogenic microbes. The respiratory burst (RB) of polymorphonuclear leukocytes (PMNs) generates an electron current that reduces molecular oxygen to superoxide. Superoxide reacts to form hydrogen peroxide as a precursor to the highly bactericidal hypochlorous acid. Here, we investigated whether alterations in extracellular potassium concentration impact H_2_O_2_ production. Such changes may occur, for example, during massive cell death due to necrosis or due to trauma injuries when potassium diffuses out of the cells. We recorded H_2_O_2_ release over a 2 h period of RB under varying potassium concentrations. Except for 100 mM potassium chloride, which increased the time delay before detectable H_2_O_2_ production, none of the potassium concentrations had a substantial effect on RB. We further examined whether this effect depended on the specific monovalent ion species. When sodium or methanesulfonate was used instead of potassium or chloride, respectively, no changes in H_2_O_2_ production were observed. Cell volume measurements under different potassium concentrations showed that only 100 mM potassium chloride significantly shrank the cells. We propose that hypertonic stress is crucial for delaying RB in human granulocytes, whereas the RB itself is independent of the tested ionic species. Additionally, the conducted hypertonic stress experiments revealed an unexpected time-dependence during the course of the RB, showing that the first 6 min were almost inert to hyperosmotic stress.

## 1. Introduction: The Respiratory Burst in Human Granulocytes

The respiratory burst (RB) in human immune cells is under close observation. It represents a profound increase in metabolism that must be fueled by carbohydrates and generates new chemical species. The essential component is the NADPH oxidase. The NADPH oxidase generates an electron current across the membrane by oxidizing NADPH and reducing molecular oxygen to superoxide. The activity of the enzyme complex depends on the assembly of its components: gp91, p47, p67, p40, p22, and Rac. The translocation of electrons resides in the transmembrane heterodimer gp91 and p22, also called NOX2, where the gp91 subunit harbors all parts needed for electron transfer, including two heme groups [1]. Consequently, the NOX2 complex has to be assembled during activation and travel to the membrane. NOX2 is preproduced in membrane vesicles that will be moved to the plasma membrane or to the sites where the foreign component may be engulfed. The trafficking of NOX2 needs rearrangement of the cytoskeleton of the phagocyte and will therefore change the shape of the neutrophil and hence the volume [2]. If the vesicles fuse with the plasma membrane, the membrane area is increased, which may cause an increase in volume. If the intracellular volume shrinks, water and ions have to leave the cytosol. Consequently, if the volume increases, water and ions have to move into the cytosol. The only way to achieve this is to move water and ions across the plasma membrane. Ion channels and pores have the highest turnover rate of all membrane transport proteins. Their turnover rate exceeds the rate of carriers, sym- and antiporters. Subsequently, one would expect ion channels to be involved in the respiratory burst in terms of volume regulation [3].

Additionally, the RB deals with charges translocated across a membrane. Electrically, the movement of charges across the membrane generates an electrical field [4], known as membrane potential. The membrane potential is one of the driving forces that drives charged particles as ions, depending on their charge, in or out of the cell. Hence, in the event of a depolarization, positively charged ions are retained less in the cytosol and increasingly driven to the extracellular space, consequently changing the osmolarity of the cell during prolonged time periods like the RB. Therefore, depending on the selectivity of the plasma membrane, ions would enter or leave the cell relative to the chemical and electrical potential. Considering that the concentrations of ions are affecting the osmolality, a cell volume decrease is expected as the efflux of ions changes the osmotic pressure inside the cell, forcing the movement of water molecules from the cytosol to the extracellular space. On the other hand, an increase in particle concentration in the cytosol would osmotically drive water back into the cell. Based on the Gedankenexperiment, it is very logical that to control the osmolarity of the cell, it is necessary to adjust cell volume and size. The membrane potential is an additional force that directly affects the cell volume by attracting or repulsing charged particles. Shrinking the cell by shape changes due to cytoskeleton compression requires movement of both water and particles–mostly ions–out. All of the above proposed actions will be fulfilled by ion channels with the benefit of not requiring additional energy from the cell, utilizing the electrochemical potential. Carriers or transporters, however, use additional energy, and their activity has to be compensated in terms of metabolic activity.

Consequently, and effectively, one would first target ion channels as potential contributors to both membrane potential changes and volume changes in the cell. Proton channels have been first suspected and later found in granulocytes [4,5,6,7,8], as the protein that compensates for the electrical activity of the NADPH oxidase, by conducting protons. However, every other cation leaving the cell would compensate for the charge of the translocated electron leaving the cell [7]. Therefore, if sodium and potassium concentrations outside the cell are increased, this might affect the RB. An increase in extracellular sodium concentration enhances the inward electrochemical driving force, facilitating sodium entry into the cell. In the case of potassium, an increased extracellular potassium concentration would decrease the chemical driving force for potassium to leave the cell and increase the electrical driving force to leave the cell. Thus, if the RB function depends to some extent on sodium or potassium extracellular concentrations, either cation should be expected to affect the RB (Figure S6). Are these increases in sodium or potassium concentration potentially taking place in the human body? In physiological terms, an increase of sodium due to an injury is less likely, rather, the main causes are dehydration or hyperaldosteronism. However, during cell necrosis, a pronounced increase in potassium is very likely. In this study we will test concentrations from 0.1 mM to 100 mM potassium in a zero potassium Ringer to investigate the effects on the RB.

## 2. Methods

### 2.1. PMN Isolation

Venous blood was drawn from healthy adult volunteers under informed consent according to procedures approved by the Institutional Review Board at the Paracelsus Medical University, Nuremberg, Germany. Blood was diluted with PBS and PMNs were purified following the publication of Boyum, 1968 [9]. PMNs were directly counted and suspended in Ringer (in mmol/L: 160 NaCl, 4.5 KCl, 2 CaCl_2_, 1 MgCl_2_, 5 HEPES, pH 7.4//Osmolarity 300 mOsmol/L). Furthermore, neutrophils were isolated using the MACSxpress^®^ Whole Blood Neutrophil Isolation Kit, human (Miltenyi, Bergisch Gladbach, Germany). The procedure was followed as described in the manual and included another 2–3 washing steps before resuspending them in Ringer. Cells were counted in trypan blue to dismiss dead cells.

### 2.2. Amplex Red Assay Quantification of H_2_O_2_ Production

H_2_O_2_ release was measured using Amplex ultra red (Invitrogen^®^ A36006; Thermo Fisher Scientific, Eugene, OR, USA), which is catalyzed by horseradish peroxidase (HRP) to the fluorescent and stable resorufin. The catalyzed reaction between Amplex Red molecules and H_2_O_2_ has a stoichiometry of 1:1, where one mol H_2_O_2_ produces one mol resorufin. Therefore, quantification of H_2_O_2_ production is possible. Measurements were performed in a volume of 100 μL, with typically 2.5 × 10^4^ cells/well. However, data normalized to one cell is displayed to increase comparability between experiments. Absolute concentrations of H_2_O_2_ were quantified from freshly prepared calibration curves in each of the experiments (Figure S5). Measurements were performed at 37 °C after brief shaking for 1 min. A dose response curve of phorbol 12-myristate 13-acetate (PMA) concentration vs. ROS production was generated. Good production and reproducibility of ROS production was achieved at a concentration of 120 nM. PMA was dissolved in 1:1 DMSO (Invitrogen) and ethanol volume (*v*/*v*). Each recording was performed in doublets to reduce possible sources of errors. The number of experiments (n) refers to independent individual experiments and does not refer to technical replicates (assay repeats with the same cells). Application of sodium and potassium was conducted by briefly stopping the H_2_O_2_ measurement and manually applying the solution by pipetting. After this break, usually less than 3 min, the measurements were continued. The time points of application are mentioned in Section 3. The maximal H_2_O_2_ production rates were determined by using the first derivative of the unprocessed data.

### 2.3. Cell Volume Measurements

Mean cell volume (MCV in fL) was measured on a Beckman Coulter Z2 particle counter (Beckman Coulter, Krefeld, Germany) based on measured changes in electrical resistance produced by non-conductive particles suspended in an electrolyte solution (Coulter method) using a 100 µm aperture tube. The upper and lower size threshold was set to 15 µm and 5 µm, respectively, and the measured volume of cell suspension per run was 0.5 mL. Calibration for particle size was performed with 10 µm Flow-Check fluorospheres (Beckman–Coulter). Data were analyzed with the Multisizer Software Ver.3.01a (Beckman–Coulter) using a 100 fL cutoff to exclude cell debris. Different samples were alternately measured in 5 min intervals from time point 0 (addition of PMA) up to 120 min, or at time points 0, 10, 20, and 40 min. Cell densities were 3–7 × 10^6^ cells/mL

All chemicals not otherwise noted were purchased from Sigma Aldrich (Sigma Aldrich, Taufkirchen, Germany).

## 3. Results

**Figure 1:**
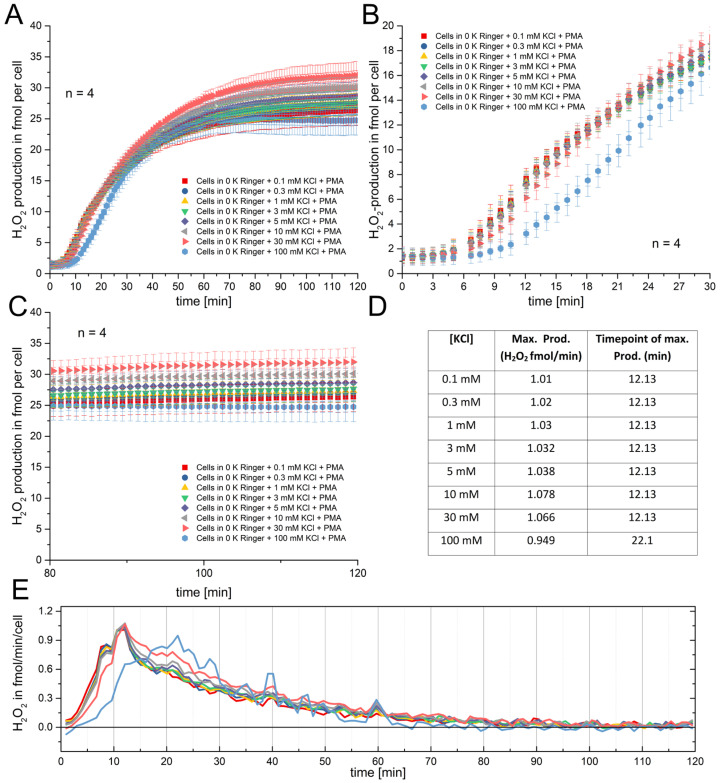
Respiratory burst (RB) measurement under several KCl concentrations. (**A**) Recording over 2 h started by the application of 120 nM PMA. Each KCl concentration in Ringer solution is depicted. The data points are the mean; the error bars are ± SD, n = 4. (**B**) Magnification of the first 30 min of the experiments. The delay introduced by 100 mM KCl is clearly visible; error bars are SD. (**C**) The final 40 min of the experiments showing almost unanimous behavior of the cells, depicted as the mean and SD. (**D**) Table showing the maximal production per cell of H_2_O_2_ and at which time point it was detected. The delay at 100 mM KCl is also visible. (**E**) The mean rate of H_2_O_2_ production plotted against the time; colors represent ionic concentrations as in panel (**A**).

First, we performed four independent experiments recording the H_2_O_2_ cumulative release over 120 min. These recordings were performed in eight different concentrations of potassium chloride (KCl) ranging from 0.1 mM to 100 mM. The pooled data is displayed in Figure 1.

The production of H_2_O_2_ over time is relatively unchanged in the first 30 min by any concentration of potassium chloride except for 100 mM. Figure 1A shows that after about 40 min, the H_2_O_2_ release levels into saturation at different values. While all tested potassium solutions show a similar start point of the RB, 100 mM potassium does introduce a delay in the RB. A close look at Figure 1B shows that at 30 mM potassium, a slight delay compared to the lower concentrations might be imagined (Figure 1, red triangles). The result is very reproducible, as the data clearly indicates. Figure 1B is the magnification of the data of Figure 1A. The delay is better visualized in this part of Figure 1. Figure 1C shows the saturation of the H_2_O_2_ production at every concentration of potassium chloride. Interestingly, it seems that the 10 and 30 mM of potassium chloride H_2_O_2_ production curves are elevated above the other concentrations. However, the variability might arise from the experimental conditions, e.g., the decrease in Amplex Red as its concentration dwindles by conversion into resorufin, and secondly, the increased variance, and therefore error bars, which does not allow specific determination of a difference at this region of the graph. The leveling of the curves, however, appears to be very consistent. Figure 1D is a calculation of the peak production of H_2_O_2_ at each of the concentrations. The peak production was determined by applying the first derivative to each of the curves (Figure 1E). All concentrations show their peak at 12.13 min, except 100 mM. The 100 mM potassium chloride concentration shifted this peak about by 10 min to 22.1 min. Curiously, the maximal rate of H_2_O_2_ production was almost unchanged, ranging around 1 fmol/min/cell.

**Figure 2:**
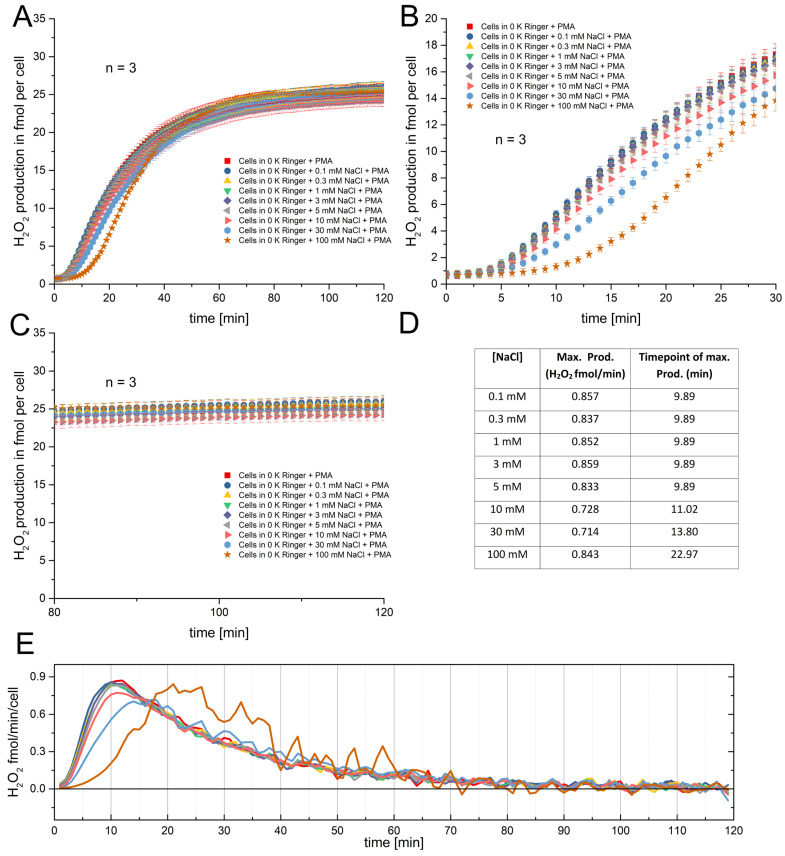
Respiratory burst (RB) measurement under several NaCl concentrations. (**A**) Recording over 2 h started by the application of 120 nM PMA. Each NaCl concentration in Ringer solution is depicted. The data points are the mean and the error bars are SE, n = 3. (**B**) Magnification of the starting 30 min of the experiments. The delay introduced by 100 mM NaCl is clearly visible, and the error bars are SE. (**C**) The final 40 min of the experiments showing almost unanimous behavior of the cells, depicted as the mean and SE. (**D**) Table showing the maximal production of H_2_O_2_ per cell and at which time point it was detected. The delay at 100 mM NaCl is also detectable here. (**E**) The mean rate of H_2_O_2_ production plotted against the time; colors represent ionic concentrations as in panel (**A**). The delays of H_2_O_2_ release are readily visible too.

The results from Figure 1 suggest that if the concentration of potassium and chloride is increased, it leads to a delay of the onset of the RB by increasing KCl to the 30 mM to 100 mM range. Nevertheless, it is uncertain if the observed effects are a consequence of either osmolarity or electrical effects. Therefore, we decided to reproduce the experiments from Figure 1 by increasing Na^+^ in equal amounts instead of K^+^. Both monovalent ions have the same charge, but they affect the membrane potential differently. In Figure 2A the same concentrations of sodium chloride were used as previously for potassium chloride, and the H_2_O_2_ production was recorded. The picture presented by the data is not much different to those presented in Figure 1. Similarly to K^+^, Na^+^ causes a noticeable delay in the onset of H_2_O_2_ production at concentrations above 30 mM (Figure 2A,B), and the saturation levels appear similar and independent of [Na^+^] (Figure 2C). The only thing which is more pronounced is the effect of the delay of 30 mM sodium chloride (Figure 2B, light blue circles). We see that the peak of production is also shifted around 12 min at 100 mM sodium chloride compared to the concentrations from 0 mM to 5 mM. The production rate of H_2_O_2_ is not as high as in the data presented in Figure 1. This might be perfectly explainable by experimental restrictions like cell counting and the calibration curve. We did another set of experiments with sodium chloride concentrations, and the rate was around 1 fmol/min, as in Figure 1.

**Figure 3:**
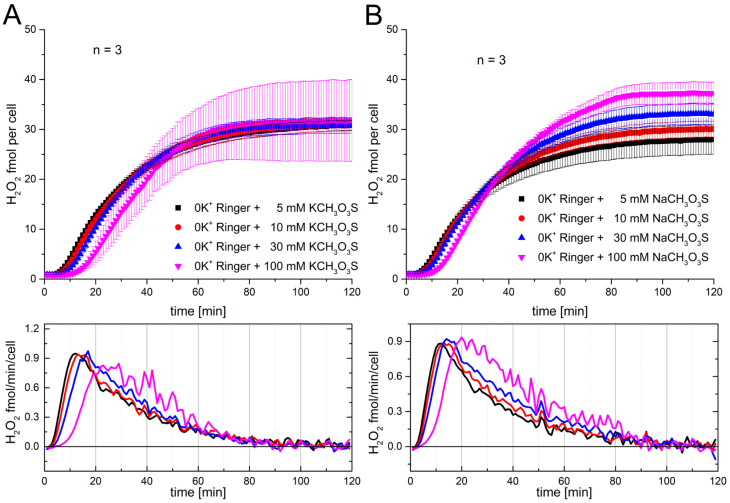
Methanesulfonate, as another anion, does not affect the RB differently to sodium or potassium. RB measurement under several KCH_3_O_3_S and NaCH_3_O_3_S concentrations. (**A**) Recording over 2 h started by the application of 120 nM PMA. Each KCH_3_O_3_S concentration in Ringer solution is depicted. The data points are depicted as the mean and the error bars are SD, n = 3. (**B**) Concentrations of NaCH_3_O_3_S affect the RB of human granulocytes comparable to KCH_3_O_3_S data, depicted as mean ± SE. Below each graph, the mean rate of H_2_O_2_ release over time is shown; colors represent ionic concentrations as in panels (**A**,**B**).

Consequently, concluding from the recorded data, it appears that the effect on the RB is independent of the species of the monovalent cation. In part this rules out that the electrical driving force is the dominant force. The very similar result for both ions on the RB proposes more an osmotic effect, independent of ionic species. To be even more specific, chloride was replaced with methanesulfonate. Methanesulfonate is an anion which is much larger in size and radius. Four concentrations of sodium methanesulfonate and potassium methanesulfonate were used. Figure 3 shows the effect of adding sodium and potassium methanesulfonate to the solution preceding the start of the RB.

Comparable to other presented recordings (Figure 1 and Figure 2), the saturation region is not as stringent as the first part of the RB. The onset shows the delay precisely at 100 mM of the added solution. There is virtually no difference between both cations and the exchange with the much bigger anion methanesulfonate. The data suggest that the main reason for the delay is based on the osmotic change that was introduced by adding higher concentrations of ions. This hypothesis is further supported by the experiments shown in Supplementary Figures S3 and S4. As shown in Figure 3, we added sucrose, an uncharged osmotic active disaccharide, which is not able to cross the cell membrane. At 200 mM sucrose, we observed a delay comparable to that shown in Figure 1, Figure 2 and Figure 3. Furthermore, we modified the ionic composition of the Ringer solution to 60 mM sodium and 100 mM potassium, maintaining an osmolarity comparable to that of the standard Ringer solution. Running these experiments in parallel showed no difference between the RB, proposing that the effect is independent of potassium or sodium ion experiments, suggesting independence from potassium or sodium ion concentration (Figure S4).

Since hyperosmotic solutions delay the respiratory burst, it is reasonable to assume that osmotic forces also affect individual cell volume. To investigate this, we measured cell volume under conditions similar to those in Figure 1. We hypothesized that attaining a specific cell volume may be required to initiate the RB. Moreover, both the restoration of cell volume in a hypertonic environment and the onset of the RB depend on metabolic activity; performing these processes simultaneously may exceed the cell’s energetic capacity.

In addition, in both cases, the cytoskeleton must adapt—both to accommodate volume loss and to restructure for the assembly and maintenance of the NADPH oxidase complex during the RB. If performing both activities simultaneously is energetically overwhelming, the cell may only be able to carry out one.

**Figure 4:**
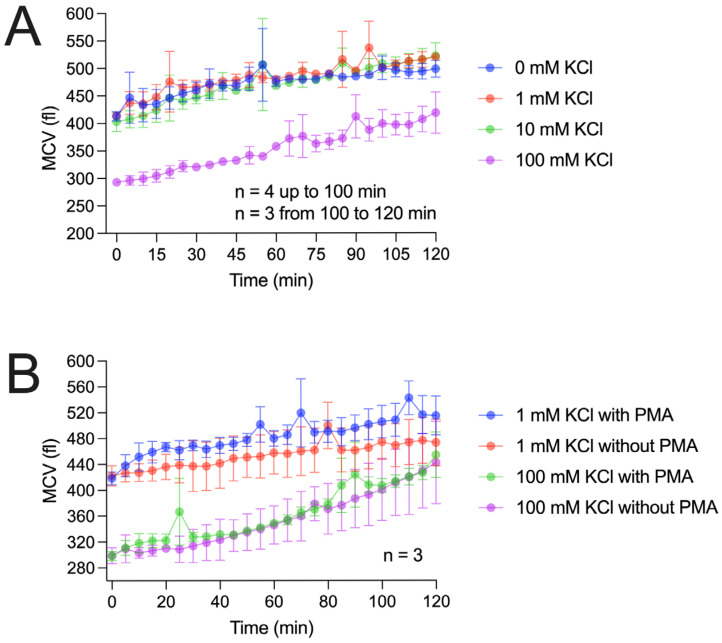
Mean granulocyte volume measurements. (**A**) Recording of the mean cell volume (MCV) of the cells in femtoliters over time. RB was triggered by PMA in four different potassium solutions. Data is depicted in mean and SD. Cell volume increased during the RB in every ionic concentration. The concentration of 100 mM reduced the cell volume, but volume increase did still take place, n = 3–4. (**B**) Comparison of the cell volume increase with or without PMA. During the first 20 min, PMA-triggered cells increase their volume faster than the control group in both tested KCl concentrations. The cell volume increase was faster in 100 mM KCl after cell shrinkage under the hypertonic conditions. Cell volume even increased in control 1 mM KCl, which might be attributed to a lack of proteins in the Ringer solution. Data in mean ± SD.

Figure 4 presents our cell volume measurements using two approaches. In the first approach, we repeated the potassium chloride experiments used to measure H_2_O_2_ production, as described in Figure 1. Similarly to the H_2_O_2_ recordings, potassium concentrations up to 10 mM did not markedly affect cell volume. However, over the 2 h recording period, cell volume increased by approximately 20% during the RB. In contrast, 100 mM potassium chloride caused a pronounced volume reduction to about 80% of the starting value. Over time, the cell volume gradually recovered (Figure 4A). Notably, after 10–15 min, cell volume had not yet returned to baseline when the RB began, arguing against the distinct cell volume hypothesis.

In the second approach (Figure 4B), we recorded cell volume changes at 1 mM and 100 mM potassium chloride, both with and without RB triggering. At both concentrations, PMA-triggered cells showed faster volume changes within the first 10–20 min compared to untriggered cells.

However, after the first 20 min, the slope of volume changes appears similar between the triggered and untriggered cells. This interesting time-dependence is also observable in the H_2_O_2_ recordings, where the first 20 min are distinct from the last 100 min of the experiment.

Based on the results, a time-dependence of the RB is proposed in the cell volume measurements, as in the H_2_O_2_ recordings.

**Figure 5:**
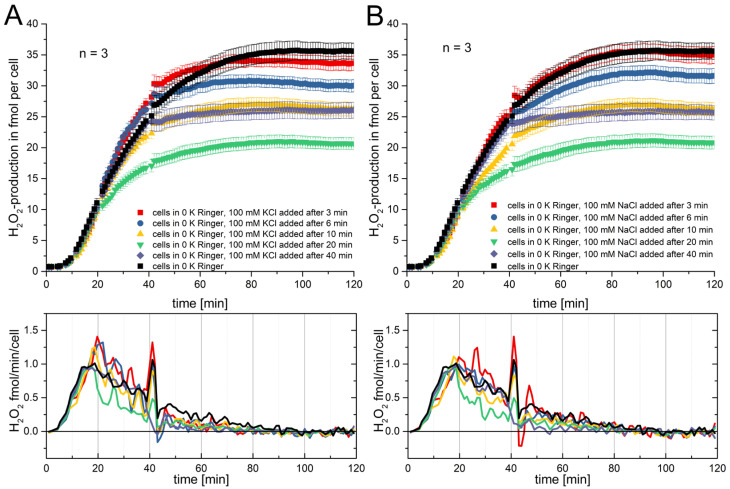
Time-dependent effects of osmotic stress during the RB. (**A**) At five different time points, 100 mM KCl was added to ongoing RB. During the first 10 min, H_2_O_2_ production remained relatively stable and unaffected by osmotic stress. After 10 min, H_2_O_2_ production progressively decreased. Addition at 20 min produced a clear effect, while addition at 40 min caused an abrupt stop. (**B**) NaCl addition showed the same time-dependent pattern, indicating independence from the cation. For both A and B: *n* = 3, mean ± SE. Below each graph, the mean rate of H_2_O_2_ release over time is shown.

The time-dependence of the RB was investigated by applying 100 mM potassium chloride or sodium chloride at distinct time points during the RB measurement. Figure 5 displays these measurements. In the experiments shown in Figure 5A, the RB was started in all wells in 0 K^+^ Ringer. Then, at 3, 6, 10, 20, and 40 min, 100 mM of potassium chloride was added. The addition at 3 and 6 min did not affect the RB visually. It did not change the H_2_O_2_ production remarkably in the first 20 min, and even up to 40 min, there are only miniscule differences. The addition at 10 min shows a slight reduction in H_2_O_2_ after 20 min. However, at the first 20 min, there is no visible effect. Yet the addition of potassium chloride at 20 min immediately impacts the RB. The production of H_2_O_2_ fully ceases in the next 20 min by showing a markedly decreased slope between 20 and 40 min. A comparable event is seen at the application at 40 min (purple trace), where the H_2_O_2_ production instantaneously stops. In Figure 5B, comparable effects in sodium chloride are measured to those seen in the potassium chloride recordings. Once more, the exchange of the monovalent cation does not strongly affect the result. There is variability in the final values of the H_2_O_2_ production, but other than that, the data is consistent. In both Figure 5A and Figure 5B, short breaks are detectable which correspond to the time points of the application of hypertonic solution. For both cations, an abrupt stop in H_2_O_2_ production is observed after applying the solution at 40 min. After 20 min, the effect on cumulative production is the strongest. Early application affects the RB little. One would hypothesize that the RB has a stable timeframe of about 10 min which is resistant against osmotic shock, while within a second time frame, the RB is vulnerable. An additional unexpected finding was that applying 100 mM potassium chloride or sodium chloride either during or before the RB produced different effects. When applied before the RB, it delayed RB onset (Figure 1 and Figure 2). However, when applied during the RB, 100 mM had no effect for approximately 10 min (Figure 5A,B; all lines except green, yellow, and purple). Thus, the ongoing RB appears resistant to osmotic disturbances.

Additionally, the same experimental design shown in Figure 5 was used to investigate the RB’s time-dependence in more detail using cell volume measurements (Figure S1). Here, the results at any time point of the application of 100 mM potassium showed cell shrinkage. Interestingly, even at the early time points (0 and 10 min) the shrinkage was not different to the application at 20 and 40 min. Thus, the mean single cell volume change does not explain the differences in H_2_O_2_ production after osmotic shock. Furthermore, comparing the histograms of the single cell volume measurements at distinct time points does not show pronounced difference in cell death compared to intact cells (Figure S2). Cell death by osmotic shock might be seen in an accumulation of very small sized debris in the histograms. In conclusion, the absolute single cell volume seems to be disconnected from the termination of the RB. If cell volume alone is not the determining factor, other mechanisms are likely responsible for the abrupt termination of the respiratory burst by hyperosmotic solutions at later time points.

## 4. Discussion

In this study we investigated the RB’s dependence on the extracellular solution. Our rate of H_2_O_2_ production by the neutrophils corresponds well with rates reported by others and us [10,11,12,13,14]. The physiological ionic concentrations in the human body are very tightly controlled. Pronounced changes in the body’s function are caused due to ionic changes. One of the best examples is a change in potassium concentration in the blood, as this leads to rhythmic and conductive problems of the heart. These changes may be so severe as to result in death.

This investigation focuses more on changes in a local environment, where changes in the local ionic concentrations derive from cell necrosis or bacterial lysis. These events may be sufficient to disturb the function of granulocytes directly, as a plethora of ion channels and transporters have been reported in these cells [2,15,16,17,18,19,20,21,22,23,24,25,26]. Most of the ion channels reported more frequently are the voltage-gated proton channel [2,5,6,7,8] and the Kir2.1 potassium channel [16,17,18]. However, it seems that our results strongly contradict the ionic effects attributed to potassium. We assume that the reported conductance, at least for potassium, is valid, as Kir2.1 channels have been reported in neutrophils and they are open at the Nernst potential for potassium [18]. However, we do not know if the Kir2.1 channels provide the dominant conductance in the plasma membrane. Therefore, the effect of the change in potassium’s Nernst potential could be lower than calculated, again showing that potassium might not strongly affect the RB via the membrane potential. In our tested conditions, doubling potassium in the extracellular solution has no effect. However, the Nernst potential would predict a change in potassium driving force by ≈20 mV, while the driving force in 30 mM K^+^ would be changed by ≈50 mV, with a barely visible effect on H_2_O_2_ production. Thus, one might doubt whether the effect of the electrical potential is the major component of the effect seen at 100 mM potassium. However, our experiments with sodium instead of potassium resulted in the same delay of the onset of the RB at comparable ionic concentrations. The Nernst potential changes by sodium would be calculated as ≈2 mV for 10 mM Na^+^ and ≈5 mV for 30 mM Na^+^. Thus, given the similarity of the delay in the RB, it is unlikely that the alterations in the Nernst potential are causing the recorded changes (Figure S6). The Nernst potential described above applies not only to potassium- or sodium-selective ion channels, but also to transporters, pumps, and nonselective ion channels. Since we tested the most abundant monovalent cations in the human body, we can likely exclude a specific dominant sodium, potassium, or nonselective monovalent conductance being activated by the RB. We tested the extracellular anion chloride by exchanging it with methansulfonate. Methansulfonate inhibits chloride channels because of its much larger size (about 2–3 times) than chloride, but with the same monovalent charge [27]. It would therefore also affect the permeability of chloride by permeating less efficiently through the pores of chloride channels in the event of a depolarization of the cell. Interestingly, the anion exchange did not lead to different results concerning the RB than those seen before, produced by the tested monovalent cations. Therefore, it seems obvious that none of the ions tested had a pronounced effect on the RB. However, an increase in the number of particles in the solution was the common pattern in all the measurements, generating higher osmolarity in the external solution (Figures S3 and S4). Water leaves the cells as the osmotic forces drive it out of the cytosol. This will lead to volume reduction in the cells according to the osmotic gradient across the cell membrane. As a rule of the thumb, we might postulate that when increasing the external solution’s osmolarity by 200 mOsmol (100 mM of NaCl or KCl), the cell’s volume should decrease to about 60% of the initial volume. This rule of the thumb implies that the cell is comparable to a giant unilamellar vesicle (GUV). This implies that there are no cellular components and everything inside the plasma membrane is filled by cytosol. The data shows a decrease to about 75% of its initial volume, which might be attributable to the fact that intracellularly in a cell there are compartments such as Golgi and the ER, reducing the cytosolic volume, or the fact that active countermeasures by the cells are in place. However, countermeasures such as actively transporting particles into the cells demand energy. The hypothesis of active countermeasures is further supported by the fact that erythrocytes are not able to withstand osmotic stress to a prolonged extent. Still, granulocytes and lymphocytes are able to withstand these conditions for much longer. This has been proven many times by lytic osmotic shock, used during cell purification to prevent the “contamination” of erythrocytes. That countermeasures are in place is evidently shown by the recovery of the cell volume in our measurements after the cells were bathed in hyperosmotic conditions. Actively transporting particles into the cell requires energy in the form of ATP. One other force that is involved during the volume changes in the cell is the membrane potential [3]. During the respiratory burst (RB), it has been suggested and demonstrated that the membrane potential undergoes depolarization [7,10,28,29,30,31]. During this depolarization, chloride ions are attracted into the cytosol by the depolarized membrane potential, which would increase the osmolarity of the cytosol. Subsequently, recovery from volume reduction would be supported by increasing the number of particles intracellularly, in this case chloride, per unit of time. Indeed, a close look at Figure 4B shows that during the first 20 min, PMA-triggered cells recover from shrinkage more quickly than untriggered cells. Evidently, depolarization is consistent with the respiratory burst of granulocytes [7]. A depolarization might be the reason for cell swelling in 1 mM KCl swell under marginal osmotic stress and even an increased rate of swelling in PMA-triggered cells. The reason why there is an overall average swelling of the cell population under little osmotic stress might be based on some cells performing a self-triggered RB. Conversely and more likely, the lack of any proteins in the Ringer solution generates some osmotic stress on the cells. Finally, it might be a simple change in the shape of the cells during the long recording times that tends to increase the volume. Are our results within the range of cell volume we would expect for neutrophils? Nibbering reported in a cell Coulter Counter a volume of 334 ± 32 fl, and Ting-Beall reports 299 ± 32 fl with video light microscopy and pipette measurements [32,33]. Our Coulter Counter reported neutrophil volumes of around 400 fl, and recent measurements on a Coulter Counter also report 400 fl [34]. Thus, our recorded data is in the range of prior cell volume measurements of human neutrophils. The volume changes we recorded in hypertonic solution also fall into the same range of data. It has to be noted that different treatments before recording, e.g., fixation of cells, leads to cell shrinkage [35]. Consequently, all cell volume data has to be critically reviewed according to the methodology used. Schmid-Schönbein [35] measured a volume reduction after changing the osmolarity from 300 mOsm/L to 575 mOsm/L to be around 15%. Ting-Beall [33] reported a volume reduction of 27% by osmolarity changes from 300 mOsm/L to 450 mOsm/L in human neutrophils. Here, our volume reduction introduced by 100 mM of KCl is in a comparable range of percentual volume loss.

Consequently, several conclusions may be drawn from our experiments. First, the RB is relatively independent from the monovalent ionic concentration changes tested in this manuscript. Physiologically, granulocytes are able to perform the RB under several ionic conditions which would surely lead to the disfunction of other cells in the same conditions. The innate immune system has a pathogen-killing mechanism (RB) which is very robust against environmental changes in the main monovalent ions in the human body. The RB’s function under extreme ionic conditions ensures its life-saving function. The RB is an electrogenic event presenting voltage amplitudes comparable to or even stronger than common action potentials but lasting much longer. Astonishingly, Na^+^ and K^+^ ions, which are crucial to the electrical activity of neurons and muscle cells, do not stop the RB in granulocytes (Figures S1–S4).

Secondly, another aspect investigated in this manuscript is the time-dependence of the RB under osmotic stress. Figure 5 shows that depending on the time point when the osmotic stress is applied, the result drastically differs between the starting and the on-going RB process. Application during the RB never results in a delay. One would consider this to be trivial, as the RB has started prior to the application of the osmotic stress. Yet, one might assume that the RB would slow down for a certain amount of time and then start again with the same rate, comparable to the result we recorded when starting the RB in hypertonic solutions (Figure 5). Interestingly, this is not the case. The RB is completely resistant to osmotic stress applied within the first 10 min. The RB responds to the osmotic stress later, a response that is first visible after 10 min. After 20 min, it visibly reduces the rate of H_2_O_2_ release, and after 40 min, the RB ceases completely. Cell volume measurements did not show that recovery from shrinkage is affected in any way by the time point of application. Thus, our investigation fails to prove the reason to explain the observed events. However, three hypotheses might be framed by the results.

1. The cytoskeleton is involved in both processes. During the RB, the NADPH oxidases have to be assembled and disassembled while it takes place. Therefore, the oxidases have to be brought to the plasma membrane and the subunits of the oxidases have to leave the membrane again. The cytoskeleton also has to adapt to the changes in cell volume. Thus, it might be impossible to complete both tasks at the same time. To conserve cell integrity, the RB would need to be delayed until the adaptation of the cytoskeleton to the new volume is completed. This delay of the onset of the RB under physiological conditions is seen in H_2_O_2_ recordings and usually spans about 3 min, readily seen in Figure 1 and Figure 2.
2. The second hypothesis is closely related to the first hypothesis, as we presume that the glucose and glycogen converted into energy might reach a maximum that would not allow both processes to take place at the same time. The RB of neutrophils produces around 1 fmol/min H_2_O_2_ at maximal rate. This converts into roughly 800,000 molecules of glucose per second into a single cell transported across the cell membrane during the RB [36]. If we assume that a glucose transporter like GLUT-1 has a turnover rate of 5000/s, we would need at least 160 transporters working at the maximal rate to achieve this transportation. However, at the same time, the cell would need energy for averting the osmotic stress and supporting actin skeleton remodeling. Solely the 800,000 molecules of glucose, if they were metabolized due to the citric cycle, would be 30,400,000 ATP missing for the cell. As there is an indication that neutrophils and eosinophils are not utilizing their mitochondria to produce ATP [37,38], one would calculate the amount of ATP via glycolysis. Eight hundred thousand molecules of glucose via glycolysis produce 1,600,000 ATP. Thus, 1,600,000 molecules of ATP are missing during the RB in an average neutrophil, as the glucose to produce this amount is used by the pentose phosphate cycle [39,40,41].
3. The removal of water from the cytosol increases proton concentration inside the cell, thereby lowering the internal pH. The exact decrease in pH is difficult to predict due to cellular buffering. Without considering buffering effects, a 25% volume reduction would correspond to an approximate pH drop of 0.1 units, or about −6 mV in the Nernst potential for protons. Although relatively small, such changes could still contribute to the observed delay of the RB.
  It seems reasonable to mention these effects, even if they arise indirectly from cell shrinkage. The pH and voltage dependence of NADPH oxidase have been previously described [4,31,42]. While the predicted shifts in pH and membrane potential are modest, they suggest that RB activation requires a certain threshold of depolarization and/or pH. Only once these parameters reach the appropriate levels does the RB trigger.

## 5. Conclusions

The RB of human neutrophils is almost independent of the extracellular free potassium concentration. Visible changes in the RB were evident under hypertonic conditions independent of the ionic species. Interestingly, the time point of the hyperosmotic solution application has an impact on the RB. Here, we report that the first 6 min are inert to the application of the hyperosmotic solution. We conclude that the RB is very stable and almost independent of potassium and sodium. However, hyperosmotic solutions can delay the RB. These results suggest that the RB works under several ionic conditions which may theoretically result from hormone shifts, massive necrosis, sepsis, cessation of blood flow, and many more. Moreover, bacterial infections and parasites may be cleared by the innate immune system in artificial non-physiological solutions, which are necessary to improve the patient’s health. One more finding in this manuscript is an intrinsic time-dependence of the RB. This time-dependence is evidently shown in this manuscript but cannot be explained. Further experiments would be needed to solve the riddle. However, carbohydrate metabolism, e.g., glycolysis, and the pentose phosphate cycle appear to be likely factors.

## Data Availability

All data is included in the manuscript or the Supplementary Materials.

## Supplementary Materials

The following supporting information can be downloaded at https://www.mdpi.com/article/10.3390/biom15101362/s1, Figure S1: Recording of the mean granulocyte cell volume upon application of hypertonic KCl solution at distinct time points; Figure S2: Granulocyte cell volume measurements displayed as histograms upon adding hypertonic KCl solution at 0, 10, 20, 40 min; Figure S3: Sucrose as uncharged but osmotic relevant particle delaying the respiratory burst in PMN; Figure S4: The H_2_O_2_ production in Ringer and modified Ringer containing increased potassium but decreased sodium concentration (same osmolarity as Ringer solution) was tested; Figure S5: Calibration curve of H_2_O_2_ in normal Ringer and in Ringer + 100 mM KCl; Figure S6: Scheme of the proposed effects on membrane potential if either potassium or sodium would be the dominate conductance in neutrophils and either sodium or potassium concentration would be increased.
