# Supplementary material for: The Respiratory Burst of Human Granulocytes Is Mostly Independent of Potassium
Source: Biomolecules. 2025 Sep 25;15(10):1362. doi: 10.3390/biom15101362 (PMC12564300; doi:10.3390/biom15101362)
Supplement: Supplementary file 1 [file biomolecules-15-01362-s001.zip › biomolecules-3724916-supplementary.pdf]

Supplementary Materials

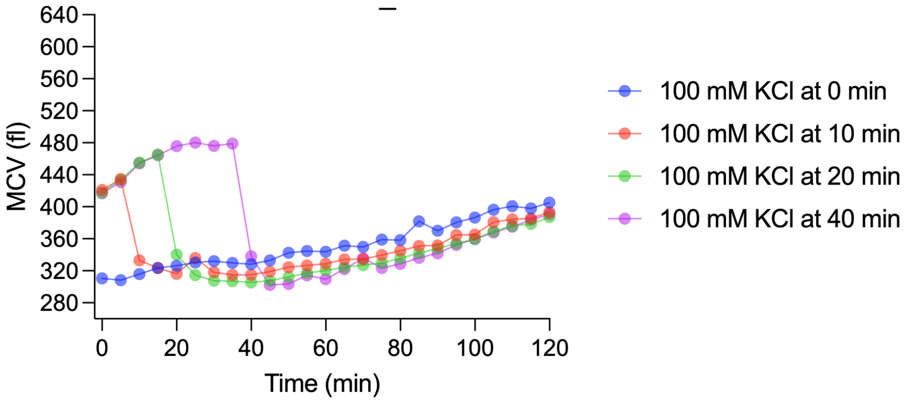

Figure S1. Recording of the mean granulocyte cell volume upon application of hypertonic KCl solution at distinct time points. Depicts a single measurement. The cells shrink in response to the hypertonic solution. No differences in cell volume are recordable nor is the volume gain after the hypertonic shock different. .

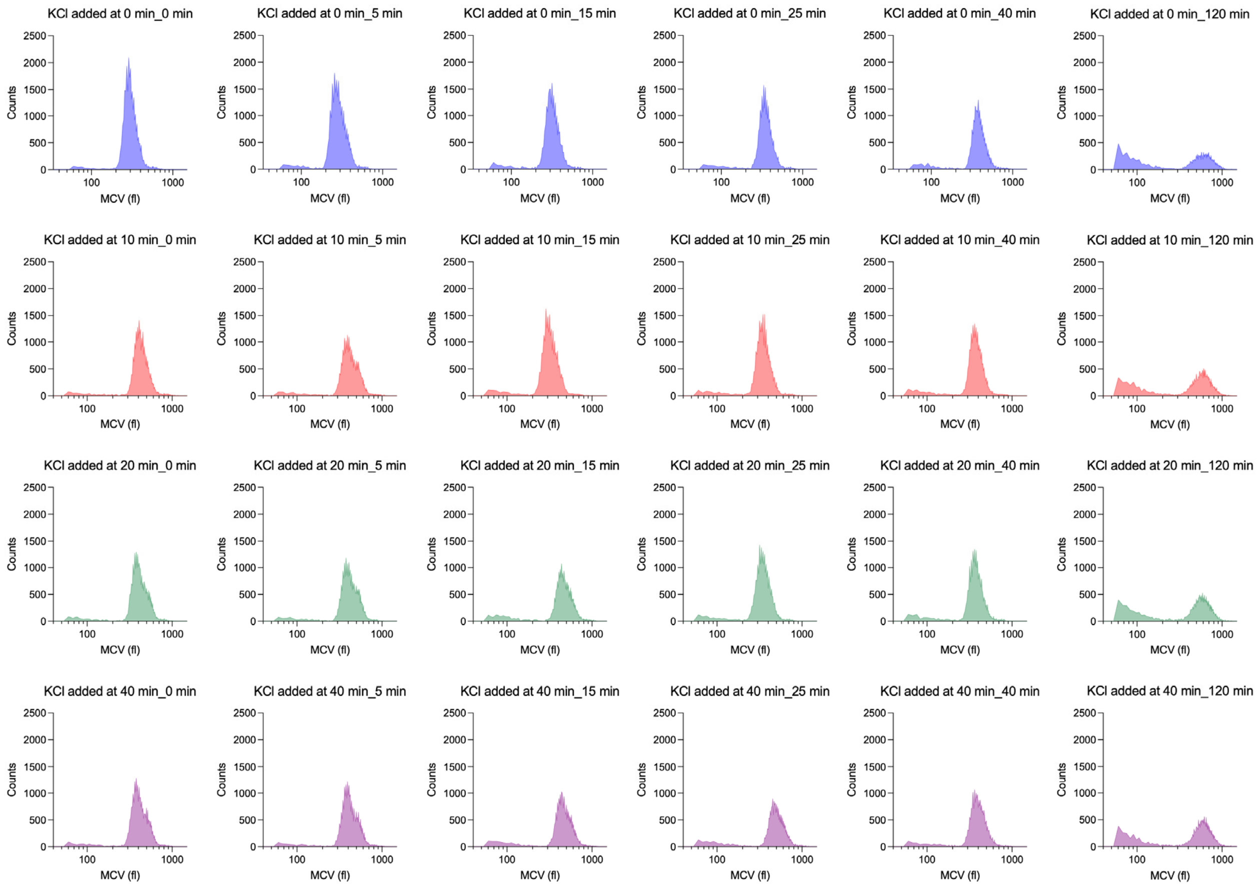

Figure S2. Granulocyte cell volume measurements displayed as histograms upon adding hypertonic KCl solution at 0, 10, 20, 40 min. Left row at 0_min in the experiment. Most right row at 120 min of the experiment. In between rows at 5, 15, 25, 45 min of the experiment. Same data as SFig. 1. The number of intact cells with a volume of > 300 fl decreases after application of the hypertonic solution. However, no immediate cell death after hypertonic solution is evident. Cells appear to degraded late in the experiment (increased debris < 220fl), but not immediately in connection to hypertonic shock.

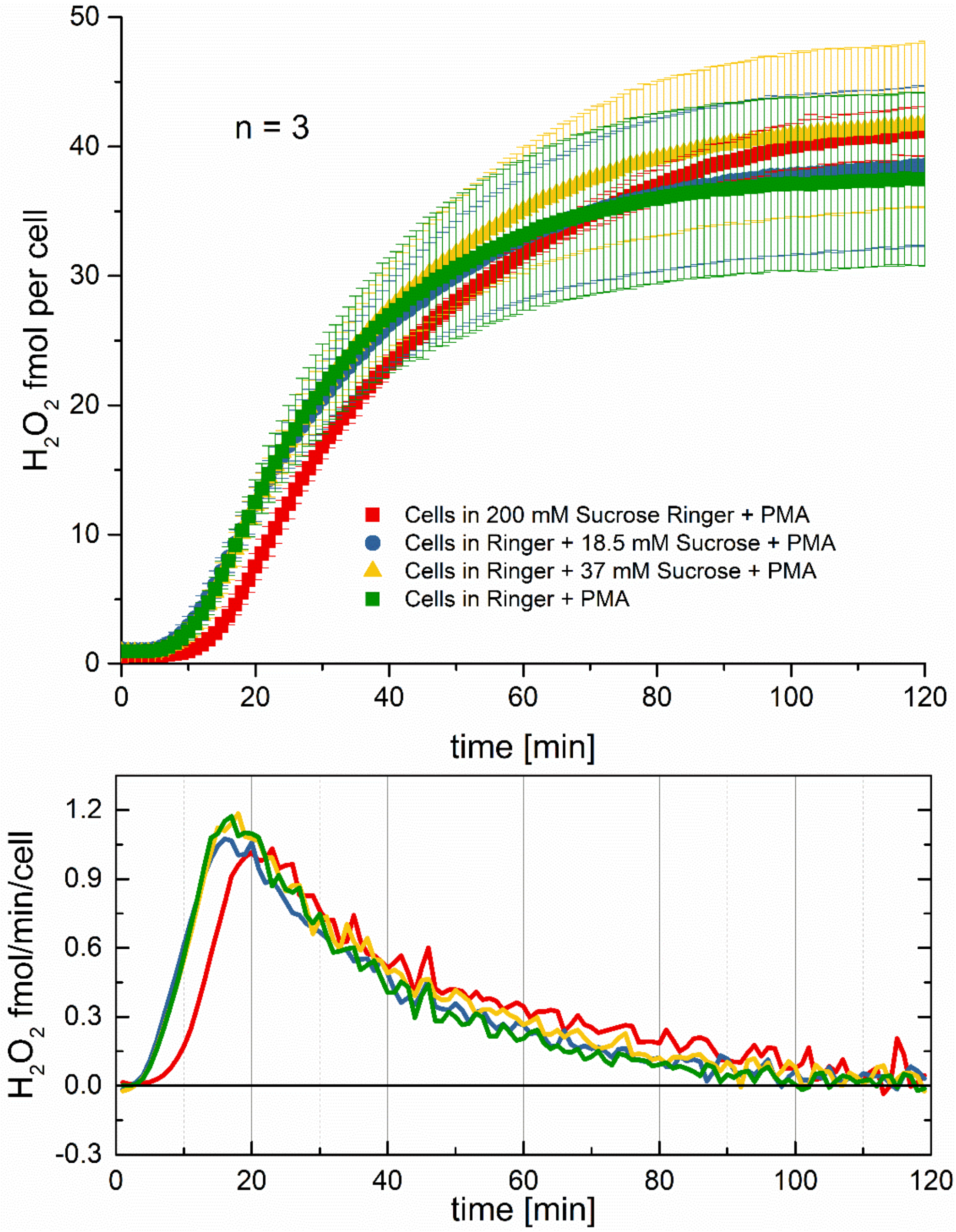

Figure S3. Sucrose as uncharged but osmotic relevant particle delaying the respiratory burst in PMN. Sucrose was added to standard Ringer 5 K^+ at 18.5 mM, 37 mM and 200 mM. Comparable to the NaCl and KCl solutions used throughout in the manuscript. Exclusively in 200 mM of Sucrose a delay was recordable. The other additions did not change the course of the experiment. Data in mean ± SD n = 3. Below mean H_2O_2 release per minute per cell over time.

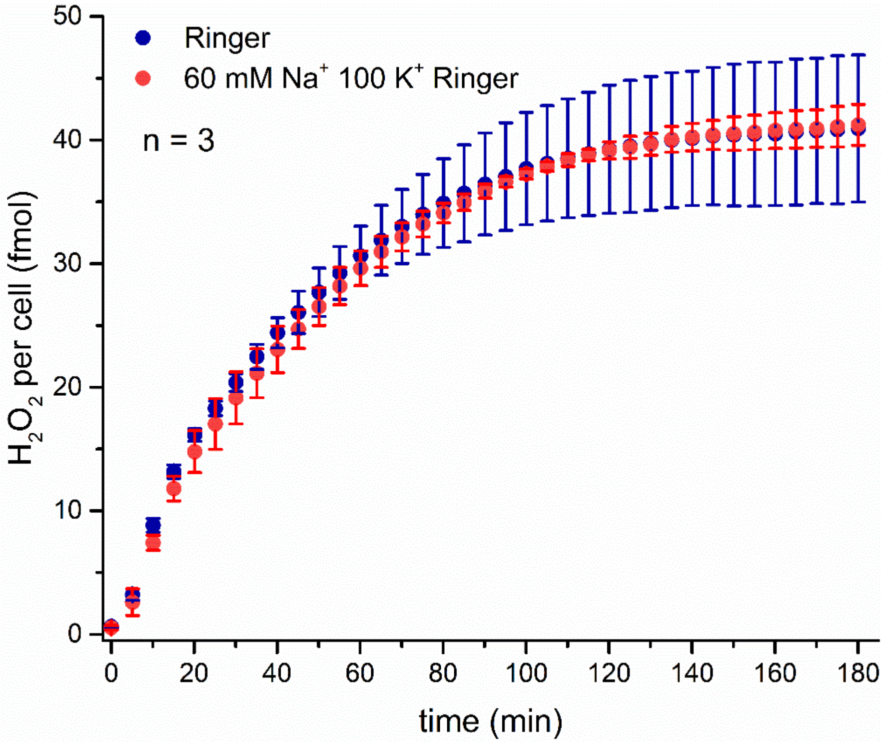

Figure S4. The H_2O_2 production in Ringer and modified Ringer containing increased potassium but decreased sodium concentration (same osmolarity as Ringer solution) was tested. Both curves overlap there is not a delay visible, normally introduced by Ringer + 100 K^+. The error bars showing the common phenomenon that at higher H_2O_2 concentrations the measurements are not as precise as in lower H_2O_2 concentrations.

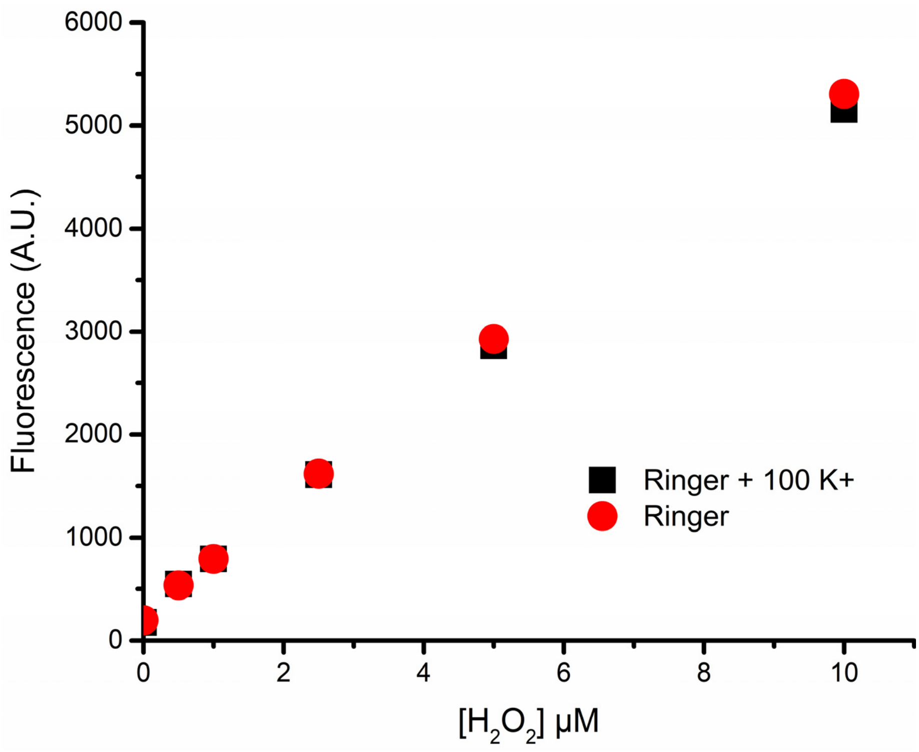

Figure S5. Above, Calibration curve of H_2O_2 in normal Ringer and in Ringer + 100 mM KCl. The data shows no direct dependence of the Amplex-Red fluorescence from the ionic conditions. Therefore, we attribute all recorded H_2O_2 changes to cellular origin. We also added the calibration curve of our experiments suggested by Reviewer 2, shown below.

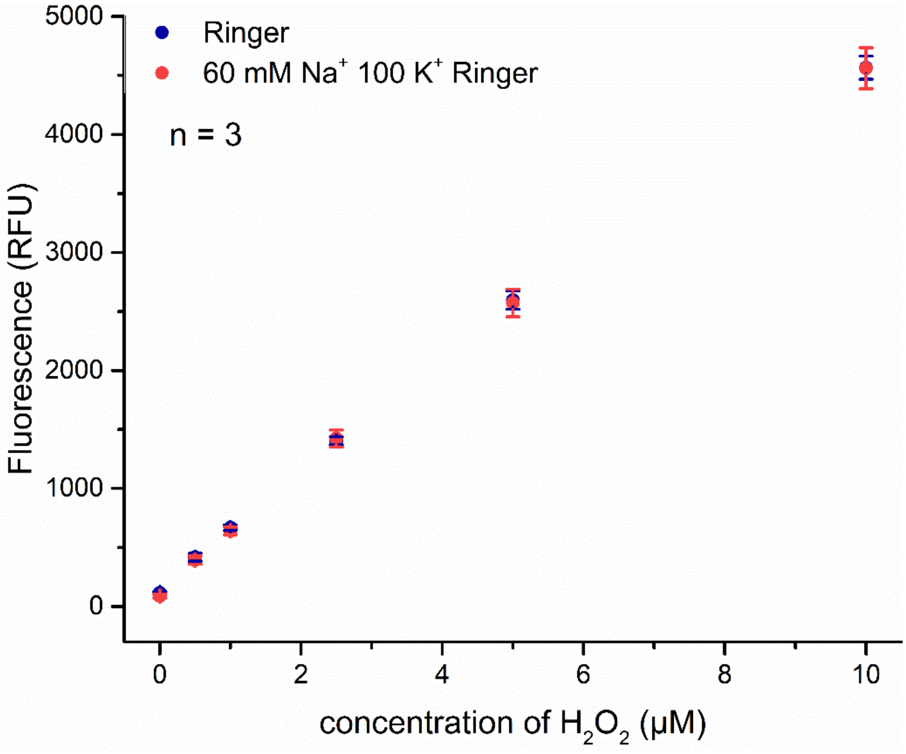

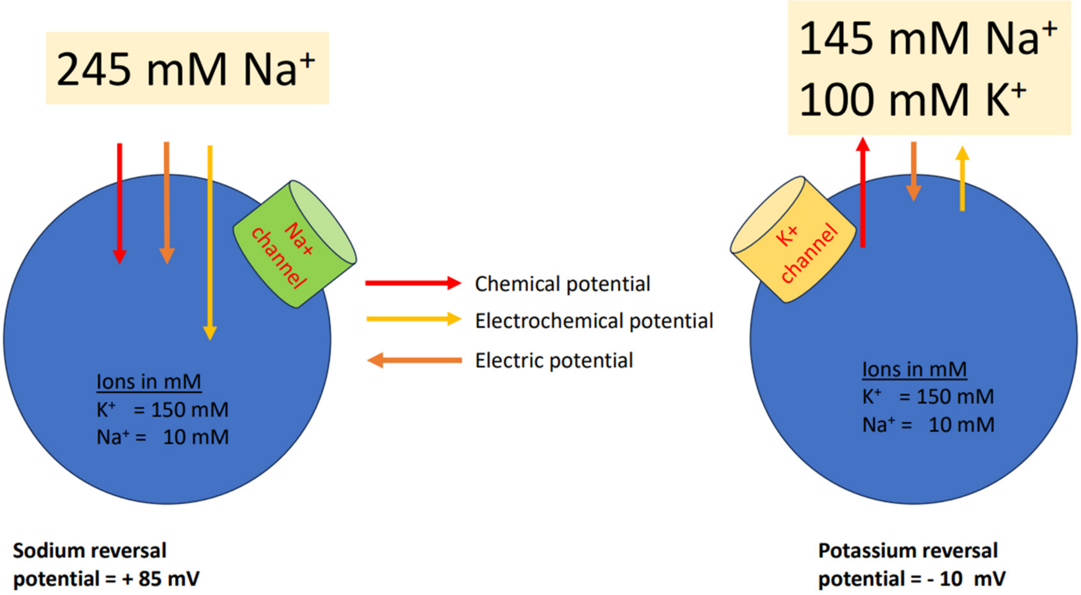

Figure S6. Scheme of the proposed effects on membrane potential if either potassium or sodium would be the dominate conductance in neutrophils and either sodium or potassium concentration would be increased.
